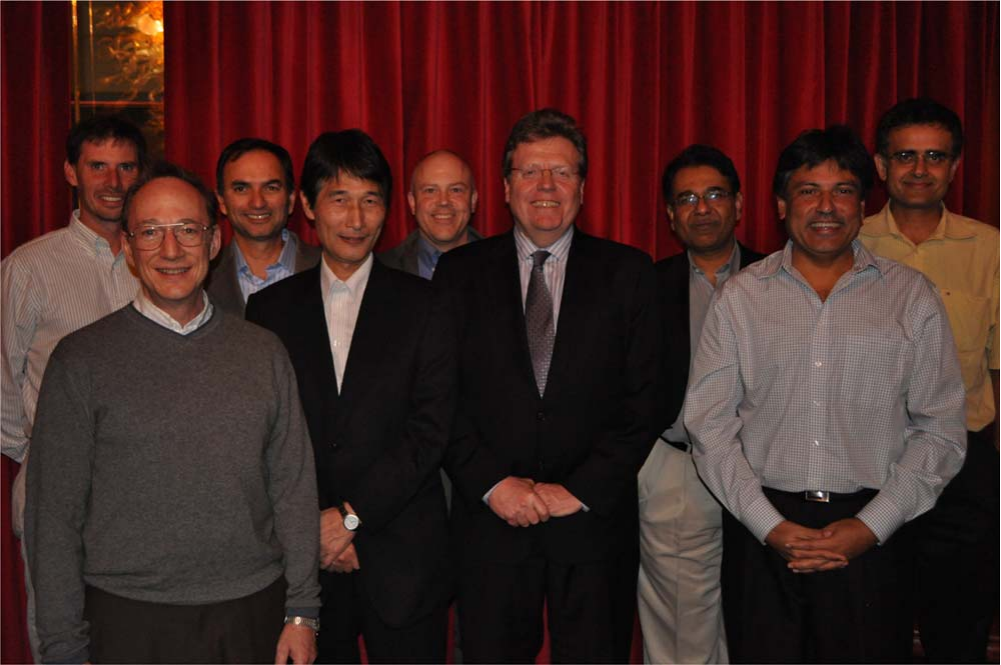# Introduction to Editorial Board Members: Professor Matthew V. Tirrell

**DOI:** 10.1002/btm2.10046

**Published:** 2016-12-16

**Authors:** Raymond Tu, James W. Schneider

**Affiliations:** ^1^ Dept. of Chemical Engineering The City College of New York ‐ CUNY New York NY 10031; ^2^ Dept. of Chemical Engineering Carnegie Mellon University Pittsburgh PA 15213

Professor Matthew Tirrell is the founding Pritzker Director of the Institute for Molecular Engineering at the University of Chicago. He received a BS degree in Chemical Engineering from Northwestern University, and he received a PhD in Polymer Science and Engineering from the University of Massachusetts.

Prof. Tirrell began his academic career at the University of Minnesota in Chemical Engineering where he earned the Shell Distinguished Chair in Chemical Engineering and established himself as a leader in the study of the polymer interfaces, adhesion, and self‐assembly. In the early 1990s, he was named the Head of the Department of Chemical Engineering at Minnesota and later held the Earl E. Bakken Chair of Biomedical Engineering while also serving as the Director of the Biomedical Engineering Institute. In 1998, he moved to the University of California—Santa Barbara to become the Richard A. Auhll Professor and Dean of the College of Engineering. After a few years as the Arnold and Barbara Silverman Chair of the Department of Bioengineering at the University of California—Berkeley, Prof. Tirrell moved to the University of Chicago to build the completely new Institute for Molecular Engineering. The Institute currently comprises sixteen faculty, with diverse research interests focused on innovative technologies in nanoscale manipulation and design at a molecular scale, with potential for societal impact in such areas as energy, health care, and the environment. Among his many talents, he has a knack for defining important scientific problems and assembling powerful, interdisciplinary groups to go about solving them.

Prof. Tirrell has been elected to the National Academy of Engineering and has been named a Fellow of the American Academy of Arts and Sciences. He is a Fellow of the American Physical Society and received both the John H. Dillon Medal and the Polymer Physics Prize from its Division of Polymer Physics. He has received several awards from *AIChE*, including the Allan P. Colburn Award, the Professional Progress Award, the Charles M.A. Stine Award, an Institute Lecturer Award, and the William H. Walker Award. He has co‐authored over 300 papers and one book, and has supervised over 80 PhD students and 40 post‐docs.

Prof. Tirrell served as the fifth Editor of the *AIChE Journal* from 1991 to 2000. His ability to work at the interface of the scientific, engineering, and medical communities both as a researcher and as an administrator has resulted in an atypical breadth of publications and awards. He is world‐renowned in two distinct areas of research: polymers at interfaces and peptide amphiphile self‐assembly. These two areas are tied together by his ability to quantitatively manipulate, measure, and thoughtfully understand the fundamental structural and biological properties of macromolecules.

Chronologically, his contributions in the area of polymer physics preceded his work with peptide‐amphiphiles. As an assistant professor at Minnesota, he began his career working on polymeric surface phenomena, making key contributions in the area of self‐healing polymeric interfaces,^1^ where his ability to quantitatively connect fundamental scaling relationships with novel experimental measurements laid the groundwork for numerous scientists and engineers working in the area. His subsequent contributions in elucidating the fundamental phase behavior of polymers confined at interfaces developed with long‐standing collaborations with Profs Lodge and Pincus in the area of polymer microstructure,^2^ Prof. Bates in the area of block co polymer phase separation,^3^ and Prof Israelachvili in the area of tribology.^4^

Prof. Tirrell made a significant departure from his earlier work in polymer physics when he began exploring the area of bioengineering and translational medicine in the 1990s. Together with Prof. Gregg Fields, he worked on the synthesis of a class of self‐assembling peptide‐based molecule called peptide‐amphiphiles.^5^, ^6^ Initially, these surface active molecules were used to create monolayers on interfaces, decorating surfaces with biologically relevant cues for cell spreading and growth.^7^ Over the subsequent two decades a number of researchers have started to use this class of molecule for a range of medical applications from tissue scaffolds to drug delivery.^8^ In doing so, Prof. Tirrell fundamentally shifted the application of peptide‐amphiphiles from a molecule for surface modification to a molecule capable of self‐assembly for translational medicine.

Recent examples include the use of peptide‐amphiphile hydrogels for peripheral nerve regeneration, where the Tirrell group synthesized peptide amphiphiles capable of co‐assembly with Type I collagen, forming mechanically stable hydrogels and quantifying the enhanced activity of Schwann cells.^9^ Moreover, the ability to use discrete self‐assembled structures as nanoparticles for drug delivery has been pioneered by the Tirrell lab. Another recent example in collaboration with Prof. Erkki Ruoslahti applies micelles as tumor targeting drug delivery vehicles, highlighting the ability to change the pharmacokinetics and decrease the systemic toxicity of a drug.^10^

As students in Prof. Tirrell's lab, we had the unique perspective of learning about the culture of research in an evolving interdisciplinary environment, where Prof. Tirrell encouraged curiosity and collaboration, creating a deep community of engaged scientists and engineers. In the Tirrell research group, Matt was always faithful to his values as an educator, passing on his sense of respect for science and all the people around him. His adventurous nature and willingness to enter new research areas has inspired dozens of his former students to pursue careers in industrial and academic research.

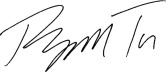


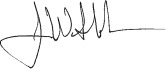

Raymond Tu^1^, James W. Schneider^2^
^1^Dept. of Chemical Engineering, The City College of New York ‐ CUNY, New York, NY 10031
^2^Dept. of Chemical Engineering, Carnegie Mellon University, Pittsburgh, PA 15213

*Matt pictured with selected PhD students and post‐docs from the 1980s. From his 60th birthday celebration at the AIChE Annual Meeting in Minneapolis*